# An inclusive approach to raising standards in general practice: working with a 'community of practice' in Western Australia

**DOI:** 10.1186/1471-2288-9-13

**Published:** 2009-02-28

**Authors:** Moyez Jiwa, Kathleen Deas, Jackie Ross, Tim Shaw, Helen Wilcox, Katrina Spilsbury

**Affiliations:** 1 Western Australian Centre for Cancer and Palliative Care, Curtin Health Innovation Research Institute, Curtin University of Technology, Perth, Western Australia, Australia; 2 Office of Postgraduate Medical Education, Faculty of Medicine, University of Sydney, Sydney, NSW, Australia; 3 General Practice, 779 Beaufort St, Mt Lawley, Perth, WA 6050, Australia; 4 Centre for Population Health Research, Curtin Health Innovation Research Institute, Curtin University of Technology, Perth, Western Australia, Australia

## Abstract

**Background:**

In this study we explored the challenges to establishing a community of practice (CoP) to address standards in general practice. We focused on the issue of improving referral letters which are the main form of communication between general practitioners (GPs) and specialists. There is evidence to suggest that the information relayed to specialists at the time of referral could be improved.

**Methods:**

We aimed to develop a community of practice consisting of GPs in Western Australia to improve the quality of referral letters to six specialty clinics. Three phases included: establishing the CoP, monitoring the progress of the CoP and sustaining and managing the CoP. The CoP's activity centred on referral letters to each of six selected specialties. A local measure for the quality of the referral letters was developed from a survey of participants about specific items of history and weighted for their perceived importance in the referral letter. Referral letters by participants written before and after the benchmarking exercise were scored for quality based on the standards set by the CoP. Feedback to participants regarding the 'quality' of their individual referrals was provided by a nominated member of the CoP, including a comparison of before and after scores.

**Results:**

15 GPs were recruited. Only five GPs submitted referral letters both before and after benchmarking. The five GPs that participated in both study phases submitted a total of 102 referral letters (53 before and 49 after). There was a 26 point (95% CI 11–41) improvement in the average scores of the second set of letters after taking clustering by speciality into account, indicating the quality of referral letters improved substantially after feedback.

**Conclusion:**

There are many challenges to forming a CoP to focus on improving a specific issue in general practice. However we were able to demonstrate that those practitioners who participated in all aspects of the project substantially improved the quality of their referral letters. For recruitment it was important to work with a champion for the project from within the practice. The project took several months to complete therefore some GPs became disengaged. Some were very disappointed by their performance when compared to colleagues. This reaction may be an important motivation to change, however it needs to be sensitively handled if participants are not to become disillusioned or disheartened.

## Background

In this study we explored the challenges to establishing a community of practice (CoP) in general practice in order to address the quality of referral letters from GPs to specialists. Etienne Wenger is credited with coining the term 'community of practice' and he defines them as "groups of people who share a concern, a set of problems, or a passion about a topic, and who deepen their knowledge and expertise by interacting on an ongoing basis." He also believes that learning is a social activity and that people learn best in groups [1]. Communities can form around a specific purpose and disband once that purpose has been achieved. Members may be very similar (e.g. general practitioners) or they may be multi-disciplinary. Some communities may be small and localised while others will be geographically dispersed 'virtual communities' that communicate primarily by telephone, e-mail, online discussion groups and videoconferencing, etc. Such communities of practice are suited to the Australian context where geography often precludes regular face-to-face meetings. This approach has been promoted as a catalyst for change in American family medicine [2]. An area or function of an organisation where knowledge is not evenly distributed is a potential target for a community of practice [3].

In Australia access to specialists is mediated by general practitioners (GPs). The process of referring a patient involves writing a letter. Patients rely on their GP to identify the at-risk profile and the specialist relies on the GP to relay that information in sufficient detail to enable the specialist to prioritise their investigations. Hodi reports that lack of information in referral letters can make it difficult to decide when cases need to be prioritised in circumstances when early access to limited resources could make a substantial difference to the outcome for the patient [4]. A report from Western Australia similarly concluded that there is a significant potential for error based on inadequate relay of information at the point of referral [5].

An example is the case of John Smith, a 46 year old man who has suffered from vague abdominal pain and diarrhoea for several weeks. This is not a real patient but one we describe for the purposes of illustration. Someone like John Smith will consult a general practitioner before he can access a specialist for investigation to establish the diagnosis [6,7]. It is now accepted that the patient with the profile described above is at significant risk of cancer and warrants urgent specialist investigations [8]. However, unless his GP mentions the duration of the symptoms or that John has a large rectal mass, or iron deficiency anaemia, or has inexplicably lost several kilograms in weight over the past four weeks, John may well be allocated an appointment in several months time by which time there may be significant progression of his underlying malignancy.

Are GPs more likely to improve their practice in this area if they are involved in a 'community of practice' with like-minded peers and agree to review their own practice? Gabbay and le May highlight the potential advantage of exploiting existing formal and informal networking as a key to conveying evidence to clinicians [9]. They describe the preference of clinicians for their own internal tacit guidelines informed largely by interactions with peers and patients and their own clinical experience. Rarely do they access evidence directly from research sources.

Glasziou and Haynes postulated that the pathway to the introduction of research evidence into clinical practice is punctuated by several steps, each of which impact on the probability that the research will be actioned [10]. Among the steps they described are three that are of particular relevance to the study described below; firstly the GP should be aware of the research evidence, secondly that she should recall that evidence when it is relevant and lastly that it should be applied. This research suggests that the exchange of information between clinicians can play an important role in facilitating evidence based practice and that the very least this exchange should not limit the application of evidence to practice. It also suggests that a CoP has the potential to increase the likelihood of the evidence being applied in practice.

## Aims

To develop a community of practice consisting of GPs in Western Australia to improve the quality of referral letters to six specialty clinics.

### Research Questions

#### Establishing and maintaining the CoP

   1. Can a CoP be established around the question of the quality of referral letters?

   2. Can the membership of such a CoP be sustained over the period of the study?

#### Monitoring the work of the CoP

   1. How can the standard of referral letters be set and monitored by the CoP?

   2. How can the performance of the CoP in relation to referral letters be shown to change over the course of the study?

#### Sustaining the CoP and managing the reaction of members

   1. How can members' interest in and reaction to feedback from the CoP be managed?

   2. How can we add value and provide closure for the CoP?

## Methods

**Figure 1:**
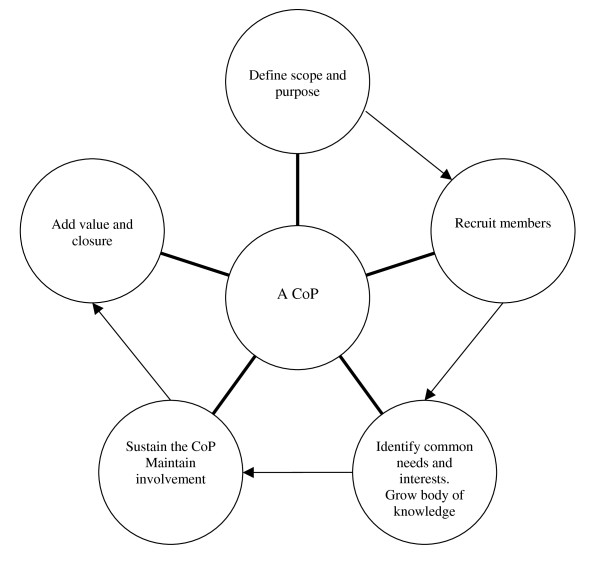
**Summary of methodology**.

Our CoP consisted of GPs working in rural and metropolitan Western Australia. The group was geographically diverse. The methodology is summarised in Figure 1 and under three headings:

### 1. Establishing and maintaining the CoP

#### Recruitment

Traditionally it is recommended to launch a CoP with a meeting or workshop so that members can meet each other and begin to develop relationships, and also spend some time together exploring and agreeing their purpose, terms of reference and ways of working [3]. However this was not practical within the geographical constraints and working schedules of the participants. Therefore recruitment took place through separate contact with individual practices and many of the participants did not meet one another either before or during the project.

The impetus for a new CoP comes from the recognition of a specific need or problem. The group was offered a very specific remit to participate in a before and after audit of referral letters, with feedback about individual performance mediated by a member of the CoP. Participation in the group acknowledged the need for action to address the issue. We clarified the specific problem that needed to be addressed (i.e. the amount of information relayed in referral letters and the specific clinical details that are pertinent in each speciality). The standards for the quality of referral letters were set by the CoP. The project involved a 'before and after' audit with peer-mediated feedback about GP referral letters written during the periods between December 2007 to February 2008 and April 2008 to June 2008. The project was reviewed by the Curtin Human Research Ethics Committee (RD-50-07) and potential participants were provided with a formal written description of what was proposed.

#### Part time practice

In some cases, such as part time GPs, the research team accepted referral letters penned on the basis of short descriptions of cases as shown in Table 1. Whilst letters based on 'vignettes' were less than ideal, it was considered better than excluding enthusiastic and motivated practitioners by virtue of not referring many patients. Some practitioners sought payment for penning 'mock' letters and local negotiations were undertaken to address this issue.

**Table 1:** Example of information provided to help construct referral letters

Patient, age [refer to]	**Problem and duration**...	**Positive findings on examination**...	**Results of Investigations: positive findings only**.....	**Other**....
Sue Eggleston, 51 [Gynaecologist]	Postmenopausal bleeding, two episodes of bleeding 6 months after menopause	Nil	Nil abnormal	Anxious, divorced from husband 3 months ago.

### 2. Monitoring the work of the CoP

As evidence for change in practice as a result of participating in this exercise, the participants were asked to set 'benchmarks' for the clinical features they considered necessary components of a GP referral letter to a specialist. Each element of history and examination were scored on a Likert scale ranging from 1, 'not important at all' to 5, 'very important'. An example of the survey and the detail of the scoring process are described on the Cancer Learning website.[11]

Most practitioners regarded each item of history and examination to be 'very important' or 'not important at all' resulting in 'skewed' responses. Few items evoked ambivalent responses. Therefore the data needed to be summarised as 'median' scores to reflect the majority view. Participants set very high standards which may have been difficult to achieve. It was important to ensure that practitioners understood that each item of history and examination that they accorded importance would need to be documented in *every *referral letter regardless of whether that information was germane to the diagnosis. The scoring of individual letters was done by a researcher with a clinical background (KD). Each element that was regarded as important in the scoring schedule was identified and ascribed the relevant score. The maximum possible score for a referral letter was 100.

Regular contact with respondents was necessary to maintain interest in the project. We also found it helpful to ensure that GPs and their practice managers received regular reminders to collect the letters for the follow up audit. We would have preferred to collect letters prospectively for both rounds of the 'audit'. However for convenience we offered to accept letters penned up to three months before the survey in order to offer feedback as soon after benchmarking as possible. The second audit involved prospective collection of referral letters from April 2008 to May 2008. It was felt that a relatively short duration for the project (6–8 weeks for collection of letters) was helpful in maintaining the momentum for the project.

#### Statistical analysis

The mean difference in referral letter scores before and after feedback for GPs who completed the study was assessed using t-tests adjusted for clustering by GP and speciality. Generalised estimating equations (GEE) were used to explore the relationship between speciality and referral scores by fitting a general linear model for the mean score that specified the within GP and within speciality correlation structure of the data. Due to the unbalanced nature of the data, mostly due to missing data from the second phase of the study, an exchangeable correlation structure and robust standard error estimation was applied. Small sample bias was addressed by scaling the robust variance estimator by n/n-p where n = number of clusters and p = number of parameters estimated. The final model included an interaction term between phase of study and speciality of referral letter. Analysis was performed using Stata Version 10.

### 3. Sustaining the CoP and managing the reaction of members

#### Maintaining members' interest and involvement

A project coordinator was employed and maintained regular email and telephone communication with the CoP. We were not able to facilitate a face-to-face meeting at any time during the project; there was no scope for social exchanges. However the group were able to challenge perspectives in the subject area by playing an active role in setting the benchmark for referral letters by participating in a postal survey. The structure and resources available to the CoP were defined. The benefits to the community were stated and the process of feedback about the 'quality' of individual performance in the project was negotiated and refined with feedback from participants. We recognised from the outset that colleagues would find this project challenging. Seldom are doctors' performances compared without the risk of implied criticism of outliers. Colleagues were therefore repeatedly reassured that their data would be handled confidentially. Referral letters were submitted with all patient identifying details removed, to preserve the privacy of patients and to avoid breaches of confidentiality.

#### Anticipating adverse reactions

After each stage of the project, follow-up letters were distributed to encourage GP feedback and to prompt the next stage in the exercise. The final style and content of the feedback was endorsed by the nominated local peer in the CoP. To maintain an inclusive approach in this exercise, the team invited comment on the format and contents of the feedback delivered to the practitioners. Every comment received by the project coordinator was acknowledged and a response drafted to explain how they helped in the project. The style of .feedback was produced over several iterations and in close consultation with the CoP. The feedback documentation included a brief guide on 'how to make sense' of the feedback. We also sent the referral letters back to their authors when giving feedback as they were unlikely to remember the letter to which the score applied.

#### Adding value and closure

Communities thrive when they are supported and valued by their host organisation, in this case the Curtin University department facilitating the project. This was a 'two-way street' so it was important that the CoP had a very clear remit from the outset rather than developing its own agenda in the course of time. This sustained the commitment to provide resources to allow the project to continue. There was a recognition and 'reward' of community members by offering comparison with peer performance relative to standards. The role of the coordinator helped to remove barriers to community membership by facilitating involvement and serving as a point of reference. Care was taken to ensure all participants were aware of the parameters for this CoP, including the timeframe. All members knew the expected outcomes and the end date for achieving them.

## Results

### Establishing and maintaining the CoP

A total of 28 practices (~70 GPs) were approached in Perth and the Greater Southern Region of Western Australia, with fifteen GPs recruited. The participants were several hundred kilometres apart. It was important at recruitment to identify a champion for the project within the practice; the practice manager fulfilled that role in most cases. The practices were contacted by phone, a personal visit, via email or post to provide information, clarify and promote the project and to encourage questions. The participants included five GPs from rural areas and ten GPs from metropolitan Perth.

### Monitoring the work of the CoP

Only five out of the 15 practitioners submitted follow up letters in the second part of the audit cycle. The demographic characteristics of the participants are shown in table 2. The mean age of full participants was 40 years, the average time in medical practice was 16.4 years, and all but one was a full time practitioner. Participants who only contributed to the first part of the audit had a mean age of 45 years and the average time in medical practice was 14 years. However there were some missing data in the latter group. Because the project took several months to complete some practitioners had become disengaged, others had forgotten to collect the letters and some had long periods of leave in the interim and had not referred any patients. Unsurprisingly, some 'short hand' items or common local abbreviation of clinical terms used in GPs' letters became apparent during the exercise and had to be clarified for scoring purposes. Feedback about the 'quality' of the GPs' individual referral letters was offered after the collection of their letters (n = 136). The referral feedback letters were distributed to the GPs (n = 15) with *each *referral letter scored according to the peer-led benchmark. Scoring required interpretation of clinical information recorded in the referrals and was consequently completed by KD with reference to a GP if necessary. To ensure consistency in our study, scoring was done by the same person for both collections of referral letters. The practitioners received feedback about each of their referral letters and their overall performance for each specialty. See Cancer Learning website for sample letters [11]. The scoring schedules prepared previously were used to .score the second set of referral letters (n = 48) and present feedback for the final phase of the project. The scoring process and values were refined based on GPs' feedback about 'shorthand' terms. Feedback letters were then distributed to the participating GPs with each referral letter scored accordingly.

**Table 2:** Demographic characteristics of participants.

GP	Age	Gender	**Location R (Rural), M (Metro)**.	Years in medical practice	Working status FT (Full time), PT (Part time) LM (Locum)	Participation in both rounds of audit cycle
1	31	F	M	8	FT	Yes

2	36	F	M	12	LM	No

3	42	F	M	20	FT	Yes

4	46	M	M	20	FT	Yes

5	38	M	M	14	FT	Yes

6	35	M	M	-	FT	No

7	34	M	M	10	FT	No

8	50	M	M	13	FT	No

9	44	M	M	20	PT	Yes

10	39	F	M	9	LM	No

11	42	M	R	15	PT	No

12	36	M	R	10	FT	No

13	29	M	R	5	FT	No

14	-	M	R	-	-	No

15	45	F	R	12	FT	No

Missing data indicated with blanks.

Information about how letters changed overall as a result of participating in the project was summarised and shared with the CoP. The final data set contained scores from 183 referral letters written by 15 GPs. Overall the average score at for the first phase was 29.3 (SD 14.1) increasing to 55.2 (SD 21.4) at the second phase out of a maximum possible score of 100. For the five GPs that participated in both study phases and submitted a total of 102 referral letters (53 before and 49 after) there was a 26 point (95%CI 11–41) improvement in the average scores of the second set of letters after taking clustering by speciality into account, indicating the quality of referral letters improved substantially after feedback. Despite only five GPs completed the study, the large number of individual referrals combined with the six different specialities meant that this study had over 95% power at an alpha level of 0.05 to detect a mean difference in before and after referral scores of 26 points with the observed intra-cluster correlation of 0.41. Linear modelling also showed that there was evidence of variation in referral letters scores by speciality (Table 3). Improvement in score was greatest for breast and respiratory referrals and least for upper GI referrals. At the end of the second phase, breast referrals had the highest mean score with those from urology, gynaecology and upper GI scoring significantly lower on average.

**Table 3:** Mean difference in referral letter scores before and after feedback.

	A			B		
Speciality	Mean Difference Phase 1 → 2	95% CI	p-value	Mean difference relative to breast referrals at second phase	95% CI	p-value
Breast	31.0	19.4–42.5	<0.001	-	-	-
Colorectal	24.8	6.7–42.7	0.006	-10.4	-21.6 – 0.7	0.065
Urology	20.4	7.3–35.6	0.002	-23.9	-38.3 – -9.5	0.001
Gynaecology	23.9	12.9–35.0	<0.001	-14.9	-20.7 – -9.1	<0.001
Respiratory	31.7	21.3–42.2	<0.001	-6.1	-14.4 – 2.24	0.153
Upper GI	16.0	4.0–28.1	0.009	-21.3	-31.2 – -11.3	<0.001

Key: A = Mean difference by specialty, B = mean difference by specialty relative to breast referrals at the second phase and after taking within-cluster correlation into account.

### Sustaining the CoP and managing the reaction of members

It was not possible to recruit new members of the CoP to replace those who could not participate in the subsequent round of the project. We would have liked to revisit the initial .survey to ensure practitioners remained satisfied with their view of the importance of elements of history and examination recommended for relay to specialists. We gleaned the impression that some participants would have adjusted the benchmarking because some elements were not as important in practice as were considered at initial survey. Members of our CoP were not asked to revisit the survey, although some participants were keen to revise their preliminary scores. Some practitioners were very disappointed and even upset by their scores. This reaction may be an important element of the subsequent motivation to change, however it needs to be sensitively handled if CoP members are not to become disillusioned or disheartened. Although many practitioners set high standards about information to be included in *every *referral letter, they were disappointed if their own letters scored poorly (when in practice they seldom record this information or consider it superfluous in most referrals). Finally we celebrated the achievements of our CoP by making a tool-kit available to others on-line.[11] We also responded to individual comments directed to the project leader.

## Discussion

### Establishing and maintaining the CoP

We report limited success with respect to establishing and sustaining membership of the CoP. The fact that the project recruited practitioners who do not normally work in the same practice or locality presented a significant challenge. It was assumed that agreement to participate was a proxy for a common interest and a common commitment to a quality agenda. This assumption could be challenged as a previous successful project on this topic recruited practitioners in the UK in an established team working in close proximity [12]. The CoP approach ideally involves at least some face-to-face meetings and some of the impetus to alter practice might be a function of social interactions and the development of a shared understanding [9]. It was not possible to facilitate face-to-face meetings within our CoP and this may have contributed to the observation that only one in three of those recruited completed the audit cycle. We also know that some practitioners were upset by their scores and may have withdrawn to spare themselves further discomfort. Secondly, the practicalities of collecting referral letters for the project may have precluded some practitioners from participating. We found that the life of the CoP needs to be limited in order to maintain momentum following establishment of a community of practice.

### Monitoring the work of the CoP

We encountered several logistical challenges in setting standards for referral letters and particularly in assessing the performance of practitioners in response to those standards. For the coordinator, based in some cases several hundred kilometres away from the participants, it was difficult to maintain contact with practice managers or GPs with busy and unpredictable schedules. It was necessary to leave messages and emails which may or may not have been relayed or given priority. The impact of any 'nuisance' factor was difficult to quantify. There were also considerable challenges to collecting the letters for the audit especially in the first part of the audit cycle where GPs had to identify letters penned several weeks or months previously. This was unexpected as it was assumed that the practice would have been able to identify referrals from their computer databases. We also perceived the need to maintain interest in the project and therefore set relatively short deadlines for completion of the second part of the audit cycle. While this may have helped the practitioners stay alert to the need to collect letters for the project, it may also have excluded those who made very few referrals.

### Sustaining the CoP and managing the reaction of members

With a high attrition rate one could argue that the practitioners either became disinterested or had other concerns about their on-going participation. Ten of the original sample of fifteen practitioners failed to complete the audit cycle. We noted some differences in their demographic characteristics although these were not striking and we are reticent to draw major conclusions from these data. However three possible reasons for this drop out exist and they may not be mutually exclusive. Firstly it is possible that the project was considered more challenging than we were able to ascertain formally. Participants set very high standards for benchmarking their referral letters. After initial feedback it may have seemed impractical to attain those standards. Secondly there were complaints during the project about the need to collect letters, in some cases manifesting as requests for funding for administrative support with this task. This support was offered on request and may have kept some practitioners involved who might otherwise have been unable to participate. The offer to fund practitioners to write 'mock' letters based on vignettes did not persuade those who declined all offers to maintain their involvement in the project. Thirdly some participants may have assumed that only referrals with a possible 'cancer' diagnosis were included in the survey and these were indeed very few. However this issue was raised early in the project and despite clarification that any referral to the relevant specialist in the second round qualified for the audit, practitioners withdrew from the project citing either a lack of referrals or lack of time.

### Limitations

There were many challenges in this study and some but not all were successfully addressed; the role of the project coordinator was critical to the success of the project. Recruitment of practitioners to what was a challenging project was facilitated by personal contact both in the metro and more especially in the rural area. However the impact of this on our final results was unquantifiable. The final tally of one in five recruited from the original sample frame was disappointing. Workload and shortage of GPs were frequently cited as reasons for declining to participate. The GP shortage is a recognised problem in the Australian context but it is not clear whether the citing this reason for declining to participate is entirely valid. [13,14]. Recruitment to research is frequently acknowledged as a major challenge to researchers in general practice. The focus of this project may not have excited the interest of many of those invited to participate. The amount of information recorded in referral letters may not be regarded by some practitioners at the coal-face as a contributor to poor service to their patients.

## Conclusion

There are many challenges to forming a community of practice around a specific issue. However in the case of those practitioners who participated in such a community of practice we were able to demonstrate an excellent response to standards set locally. Participation in such a project based on an agenda or issue identified externally to the CoP may result in ambivalence towards the project. The next step may be to form a CoP around a subject that more broadly appeals to each member of the CoP. However there is a danger that practitioners may not participate in projects where there are potentially embarrassing low standards. The logistics of maintaining a CoP across a wide geographical area are considerable and the focus of any future project therefore warrants particular attention.

## Competing interests

The authors declare that they have no competing interests.

## Authors' contributions

MJ, JR and TS designed the study and co-authored the paper. HW participated in the study and co-authored the paper. KS analysed the data and co-authored the paper. KD coordinated the study, entered the data and co-authored the paper. All authors read and approved the final manuscript.

## Pre-publication history

The pre-publication history for this paper can be accessed here:

http://www.biomedcentral.com/1471-2288/9/13/prepub
